# INDEPENDENT STRATUM FORMATION ON THE AVIAN SEX CHROMOSOMES REVEALS INTER-CHROMOSOMAL GENE CONVERSION AND PREDOMINANCE OF PURIFYING SELECTION ON THE W CHROMOSOME

**DOI:** 10.1111/evo.12493

**Published:** 2014-08-29

**Authors:** Alison E Wright, Peter W Harrison, Stephen H Montgomery, Marie A Pointer, Judith E Mank

**Affiliations:** 1 Department of Zoology, Edward Grey Institute, University of OxfordOxford, OX1 3PS, United Kingdom; 2 Department of Genetics, Evolution and Environment, University CollegeLondon, London, WC1E 6BT, United Kingdom

**Keywords:** Chromosome inversion, female heterogamety, recombination suppression

## Abstract

We used a comparative approach spanning three species and 90 million years to study the evolutionary history of the avian sex chromosomes. Using whole transcriptomes, we assembled the largest cross-species dataset of W-linked coding content to date. Our results show that recombination suppression in large portions of the avian sex chromosomes has evolved independently, and that long-term sex chromosome divergence is consistent with repeated and independent inversions spreading progressively to restrict recombination. In contrast, over short-term periods we observe heterogeneous and locus-specific divergence. We also uncover four instances of gene conversion between both highly diverged and recently evolved gametologs, suggesting a complex mosaic of recombination suppression across the sex chromosomes. Lastly, evidence from 16 gametologs reveal that the W chromosome is evolving with a significant contribution of purifying selection, consistent with previous findings that W-linked genes play an important role in encoding sex-specific fitness.

Recombination suppression initiates the divergence of sex chromosomes from an ancestral autosomal pair, and promotes the degeneration of the W (in female heterogamety) and Y (in male heterogamety) chromosome coding content (Ohno 1967; Charlesworth 1991; Charlesworth and Charlesworth 2000; Charlesworth et al. 2005; Bachtrog et al. 2008). In spite of the pivotal role recombination suppression plays in sex chromosome evolution, our understanding of the underlying mechanisms and dynamics is largely limited to either highly divergent sex chromosomes (e.g., mammals and *Drosophila*), or incipient sex chromosome systems, such as those observed in plants and fish (Chibalina and Filatov 2011; Muyle et al. 2012; Bergero et al. 2013; Natri et al. 2013; Qiu et al. 2013).

Older sex chromosomes support the classic model of sex chromosome evolution, where intra-chromosomal rearrangements are proposed to initiate the suppression of recombination across large regions (Charlesworth et al. 2005). Theoretically, these inversions result in instantaneous recombination suppression between the sex chromosomes. This leaves a spatial signature of clusters of gametologs, orthologous genes on the X and Y (or Z and W) chromosomes, of similar divergence, often referred to as strata. Strata are thought to form independently in a stepwise process over millions of years. At least four chromosomal rearrangements occurring over 300 million years have been documented on the human X and Y chromosomes (Lahn and Page 1999; Ross et al. 2005; Lemaitre et al. 2009; Wilson and Makova 2009; Pandey et al. 2013; Bellott et al. 2014), and there is evidence of four strata on the avian sex chromosomes spanning 130 million years (Wright et al. 2012).

Newly evolved sex chromosomes show less concordant support for the strata model. The threespine stickleback (*Gasterosteus aculeatus*) and white campion (*Silene latifolia*) X and Y chromosomes originated around 30 million years ago (Mya) and less than 10 Mya, respectively. Work on these systems indicates that recombination suppression evolves heterogeneously along the length of the chromosome (Chibalina and Filatov 2011; Muyle et al. 2012; Bergero et al. 2013; Natri et al. 2013; Qiu et al. 2013; Roesti et al. 2013). This suggests that although selection to suppress recombination between the X and Y chromosomes occurs over large regions, within those regions, the effects are variable and genetic exchange between the sex chromosomes persists in some places.

We need to fully integrate short- and long-term views of sex chromosome evolution to provide a complete temporal overview of the dynamics of recombination suppression. Additionally, much of our understanding of sex chromosome evolution is based on studies of neo-sex chromosomes in *Drosophila* (Bachtrog et al. 2008; Kaiser et al. 2011; Zhou and Bachtrog 2012). These have been highly informative, however, males are achiasmatic in *Drosophila* and therefore cannot be used to study progressive recombination suppression events. Instead, comparative analyses of sex chromosome divergence across a range of species with recombination in both sexes are required to provide an evolutionary framework to characterize the temporal dynamics of sex chromosome evolution.

The avian sex chromosomes are homologous across the entire clade, however recombination suppression between the Z and W has evolved repeatedly in different lineages across 130 million years (Tsuda et al. 2007; Suh et al. 2011). We use the repeated evolution of avian sex chromosome strata to create a cohesive view of short-, medium-, and long-term dynamics in Z–W divergence patterns. To do this, we assembled the largest cross-species dataset of W-linked coding content to date spanning the Galliformes (the landfowl) and the Anseriformes (the waterfowl). These two sister orders, the Galloanserae, last shared a common ancestor approximately 90 Mya (van Tuinen and Hedges 2001). Our analysis indicates that the majority of the Z and W chromosomes formed independently in each of these orders. Additionally, our results suggest ongoing recombination between gametologs in the most recent region of the Anseriform sex chromosomes, and gene conversion throughout the sex chromosomes.

## Materials and Methods

### IDENTIFICATION OF W-LINKED GENES

Previous efforts to identify W-linked sequences in birds (Chen et al. 2012) have resulted primarily in noncoding sequence, which makes comparisons to the Z chromosome difficult. To expand the known coding content of both the mallard duck (*Anas platyrhynchos*) and wild turkey (*Meleagris gallopavo*) W chromosomes, we used a combined approach based on RNA-seq data and sequence similarity to known red jungle fowl (*Gallus gallus*) W-linked genes.

RNA-seq data were obtained from captive populations of *A. platyrhynchos* and *M. gallopavo*, taken at the start of their first breeding season (year 1 for *A. platyrhynchos*, year 2 for *M. gallopavo*). All samples were collected in accordance with national guidelines and with permission from institutional ethical review committees. The left gonad was collected separately from five male and five female *A. platyrhynchos*, and seven male and five female *M. gallopavo*. The spleen was collected from five male and five female *A. platyrhynchos* and four male and two female *M. gallopavo*. The samples were homogenized and stored in RNAlater until preparation. We extracted RNA using the Animal Tissue RNA Kit (Qiagen) and The Wellcome Trust Centre for Human Genetics, University of Oxford, prepared and barcoded samples using standard methods. RNA was sequenced on an Illumina HiSeq 2000 resulting in on average 26 million 100 bp paired-end reads per sample.

The data were assessed for quality using FastQC version 0.10.1 (www.bioinformatics.babraham.ac.uk/projects/fastqc) and filtered using Trimmomatic version 0.22 (Lohse et al. 2012). Specifically, reads with residual adaptor sequences were removed and reads were trimmed if the leading or trailing bases had a Phred score <4 or the sliding window average Phred score over four bases was <15. Post filtering, reads were removed if either read pair was <25 bases in length. Filtered reads from each species were mapped to the respective reference genomes obtained from Ensembl version 74 (Flicek et al. 2013; *M. gallopavo* version 2.01/GCA_000146605.1; Dalloul et al. 2010; Dalloul et al. 2014) and *A. platyrhynchos* version 1.0/GCA_000355885.1; Huang et al. 2013) using TopHat2 version 2.09 (Trapnell et al. 2009; Kim et al. 2013) and Bowtie2 version 2.1.1 (Langmead et al. 2009; Langmead and Salzberg 2012). For recently evolved gametologs with high sequence similarity, it is possible that Z-linked reads will spuriously map to W-linked exons. To avoid this and allow accurate identification of female-limited genes and therefore putative W-linkage, both reads of a pair had to map concordantly to the reference sequence and no mismatches in the alignment were permitted. This strategy, although essential to accurately differentiate gametologs, could fail to identify W-linked genes when the entire locus is not encompassed on a single scaffold in the genome assembly.

To identify polymorphisms between Z and W coding sequences the mapping criteria were relaxed. For each sample, we used Cufflinks version 2.1.0 (Trapnell et al. 2010, 2013) to estimate transcript abundances for Ensembl annotated genes as fragments per kilobase per million mappable reads. We identified strongly female-biased and female-limited genes as putative W-linked genes using gene expression profiles in males and females (Fig. S1).

Putative W-linked genes were also identified by high sequence similarity to known G. gallus W-linked genes. *Gallus gallus* (Galgal4.0/GCA_000002315.2), *M. gallopavo*, and *A. platyrhynchos* cDNA sequences were obtained from Ensembl version 74 (Flicek et al. 2013) and for each species the longest transcript for each gene was identified. We determined putative orthology using BLAST version 2.2.26 (Altschul et al. 1990) with an e-value cutoff of 1 × 10^−10^ and identified putative *A. platyrhynchos* and *M. gallopavo* W-linked genes that had the highest BLAST score to known *G. gallus* W-linked genes.

For both *A. platyrhynchos* and *M. gallopavo*, we validated putative W-linked genes by PCR using genomic DNA from three individuals of each sex.

### IDENTIFICATION OF GAMETOLOGS

**Figure 1:**
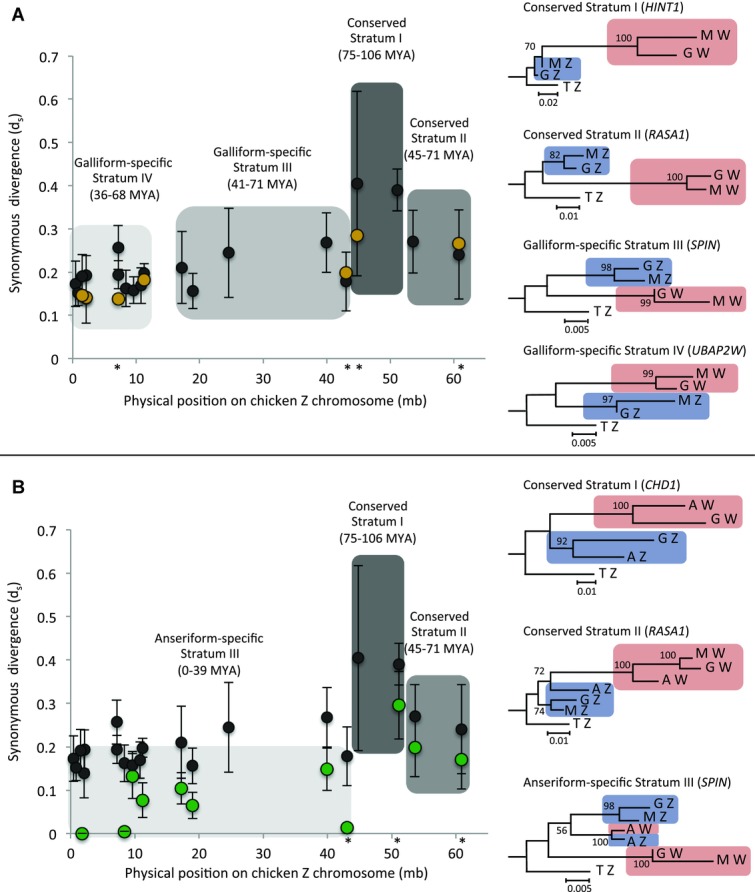
Dynamics of recombination suppression on the avian sex chromosomes. The distribution of synonymous (*d*_S_) divergence estimates between *G. gallus* (black), *M. gallopavo* (yellow, panel A) and *A. platyrhynchos* (green, panel B) gametologs, mapped against physical position of the *G. gallus* Z-linked ortholog. The 95% confidence intervals were calculated using standard errors. Bootstrap values were calculated using 1000 permutations. AZ, GZ, MZ, TZ corresponds to *A. platyrhynchos*, *G. gallus*, *M. gallopavo*, and *T. guttata* Z-linked genes. The physical Z-linked position of the loci for which gene trees are shown are indicated (*). *Meleagris gallopavo d*_S_ estimates map closely onto the *G. gallus* strata boundaries, and maximum-likelihood gene trees for all gametologs cluster by sex chromosome, not species, indicating that four strata are conserved across the Galliformes (Fig. S2). *Anas platyrhynchos d*_S_ estimates map closely onto the two oldest *G. gallus* strata boundaries, and maximum-likelihood gene trees for gametologs within these regions cluster by sex chromosome indicating that these strata are conserved across the Galloanserae. For the remaining gametologs, maximum-likelihood gene trees reveal that recombination was suppressed independently in the *A. platyrhynchos* lineage (Fig. S3). The *d*_S_ estimates are highly heterogeneous and locus specific, indicating gradual divergence of gametologs with residual recombination across large tracts of the sex chromosomes.

Previously, we used newly identified *G. gallus* gametologs to identify four strata on the Z chromosome (Wright et al. 2012). Subsequent to this work, the Ensembl annotation of the *G. gallus* W and Z chromosomes has been revised (Flicek et al. 2013) and additional W-linked genes have been identified (Ayers et al. 2013). To account for these changes, we updated gametologs and reanalyzed divergence estimates using coding sequences from Ensembl version 74 (Flicek et al. 2013) and PAML version 4.7 (Yang 2007). Revised divergence estimates were compared to previous estimates (Wright et al. 2012) with Spearman's correlation in the rcorr function (Hmisc package [Schemper 2003; Tian et al. 2007] in R [R Core Team 2011]). Across gametologs, revised *d*_S_ estimates were strongly correlated with previous estimates from Wright et al. 2012 (Spearman's rank correlation *P*-value < 0.01, correlation coefficient 0.78). The *d*_S_ of three newly identified gametologs were also consistent with the previously identified strata, with overlapping 95% confidence intervals (Fig.1, Table S1), in line with the previous findings that the *G. gallus* Z chromosome was formed by four independent recombination suppression events.

*Anas platyrhynchos, M. gallopavo*, *G. gallus*, and *T. guttata* (taeGut3.2.4) coding sequences were obtained from Ensembl version 74 (Flicek et al. 2013), and additional *M. gallopavo* sequences were obtained from Genbank (Backstrom et al. 2005; Berlin and Ellegren 2006). For each species the longest transcript for each gene was identified. *Gallus gallus* sequences were BLASTed reciprocally against *A. platyrhynchos, M. gallopavo*, and *T. guttata* coding sequences using an e-value cutoff of 1 × 10^−10^ and the highest BLAST score was used to identify the best BLAST hit.

For *A. platyrhynchos* and *M. gallopavo* W-linked genes, we identified orthologous Z-linked genes using the following steps. For each W-linked gene (1) we identified the orthologous *G. gallus* W-linked gene using BLAST and the *G. gallus* Z-linked ortholog from Wright et al. (2012); (2) *A. platyrhynchos* and *M. gallopavo* Z-linked orthologs were defined as the reciprocal best hit of the *G. gallus* Z-linked ortholog; (3) in a few instances there was no reciprocal best hit, often because of high sequence similarity between Z and W gametologs. For these genes, the Z-linked reciprocal ortholog was identified as the next best BLAST hit of the *G. gallus* Z-linked ortholog.

Avian genomes are particularly stable with few major genomic rearrangements (Stiglec et al. 2007) and little gene movement off and onto the avian Z chromosome, potentially due to a lack of active transposons (Toups et al. 2011). We therefore expect Z-linkage and synteny to be highly conserved across *A. platyrhynchos, M. gallopavo*, and *G. gallus*. We verified Z-linkage of reciprocal orthologs using positional information in the *M. gallopavo* genome assembly. The *A. platyrhynchos* genome assembly lacks annotated chromosomes and as recently diverged Z- and W-linked genes share a high degree of sequence similarity, to avoid misidentifying the Z-linked reciprocal ortholog as a W-linked paralog, we verified the reciprocal ortholog was not located on the W chromosome using PCR. However, this approach does not distinguish Z chromosome to autosomal gene duplication, but given the pronounced stability of avian genomes (Toups et al. 2011), this is unlikely to bias our results.

### DIVERGENCE OF GAMETOLOGS

To identify the number and boundaries of evolutionary strata in *A. platyrhynchos* and *M. gallopavo*, we estimated rates of synonymous divergence (*d*_S_) (Wright et al. 2012), as *d*_S_ provides a relative measure of the relative length of time gametologs have differentiated from an ancestral autosomal gene. For each species, we obtained Z and W coding sequences from Biomart, and aligned the translated protein sequences with MUSCLE (Edgar 2004a,2004b) in MEGA5 (Tamura et al. 2011). Alignments were visually inspected and poorly aligned regions were removed. The CODEML package in PAML version 4.7 (Yang 2007) was used to calculate maximum-likelihood estimates of synonymous (*d*_S_) and nonsynonymous (*d*_N_) divergence in pairwise comparisons and generate standard errors. We verified *d*_S_ ≤ 1 for all gametologs, thereby avoiding inaccurate divergence estimates due to mutational saturation and double hits (Axelsson et al. 2008).

Three *A. platyrhynchos* gametologs had *d*_S_ < 0.02. We verified that the annotated Z and W genes were distinct coding sequences by identifying female-limited SNPs using RNA-seq data and W-linked SNPs from Ensembl annotated sequences.

Some gametologs had multiple W paralogs, and to identify the true W-linked ortholog we used the method outlined in Wright et al. (2012). Briefly, we used the gametologous pair with the lowest *d*_S_ estimate to avoid potential problems of relaxed or diversifying selection acting on gene copies (Graur and Li 2000) likely due to gene duplications following the suppression of recombination (Busby et al. 2011). However, the *A. platyrhynchos* draft assembly contains short sequences that may represent partial fragments of longer W-linked genes. To avoid biasing divergence estimates on short stretches of sequence, we excluded W-linked genes if coverage of the *G. gallus* W-linked ortholog was <25% and the aligned length with the Z-linked ortholog was <150 bp. This resulted in the removal of three *A. platyrhynchos* W-linked genes from the analysis. All *M. gallopavo* W-linked genes met these criteria, and therefore none were excluded.

For each species, we mapped *d*_S_ estimates to the Z chromosome, taking physical position from the orthologous *G. gallus* Z-linked ortholog. Divergence dates for gametologs were calculated using a molecular clock. Briefly, based on male-mutation bias estimates in birds, the molecular clock accounts for sex-specific mutation rates in Z- and W-linked genes. This results in a divergence time of *d*_S_/(3.8 × 10**^−^**^9^) years (Dimcheff et al. 2002b; Hedges 2002; Harlid et al. 2003; Axelsson et al. 2004; Berlin et al. 2006; Webster et al. 2006; Nam and Ellegren 2008).

We used *M. gallopavo* gametologs to independently confirm the four strata previously identified in *G. gallus* (Wright et al. 2012) and to refine the dates at which recombination was suppressed. We assessed longer term dynamics of sex chromosome divergence using *A. platyrhynchos* gametologs. To do so, we built gene trees to determine whether recombination ceased independently between gametologs in each species, or before the last common ancestor of the Galloanserae. Specifically we (1) obtained coding sequences for gametologs in the *G. gallus*, *A. platyrhynchos, M. gallopavo*, and *T. guttata* from Biomart, and aligned the translated protein sequences with MUSCLE (Edgar 2004a, b) in MEGA5 (Tamura et al. 2011). We checked alignments by eye and removed poorly aligned regions. *Taeniopygia guttata* Z-linked orthologs, identified from a reciprocal BLAST with *G. gallus*, were used as outgroups; (2) we constructed maximum-likelihood gene trees with 1000 bootstrap replicates in MEGA5 (Tamura et al. 2011). In the absence of overlapping sequences, gene trees were constructed between *G. gallus* and *A. platyrhynchos* or *M. gallopavo* gametologs separately. In all cases, the *T. guttata* Z-linked ortholog was designated as the outgroup.

To measure the completeness of recombination suppression between gametologs, we tested for evidence of gene conversion using GENECONV (Sawyer 1999). GENECONV identifies potential gene conversion sites as long fragments of identical sequence bounded by variable sites, assessing significance by 10,000 random permutations of variable sites across the alignment. *P*-values for global fragments are corrected for multiple comparisons and sequence length. We tested for gene conversion using the multiple sequence alignments described above (used to build gene trees) and specified the *–group* option to look only for conversion between gametologs in *G. gallus*, *A. platyrhynchos*, and *M. gallopavo*. We used the –Seqtype = SILENT option to specify coding sequence and that gene conversion fragments should only be detected using silent site amino acid polymorphisms. High false-positive rates have been documented when this option is not implemented, particularly if genes are subject to contrasting selective regimes (Ezawa et al. 2006, 2010). We used the strict default *-gscale* value (*gscale* = 0) where no mismatches are permitted between aligned putative conversion fragments and repeated the analysis with a relaxed mismatch penalty (*gscale* = 2) to ensure the number of mismatches did not bias our analysis. GENECONV detects inner fragments, which arise from gene conversion events between the ancestors of two aligned sequences and outer fragments, which represent a conversion events originating outside of the alignment or events whose signature has been weakened by subsequent mutation. We identified significant inner gene conversion fragments between gametologs as evidence of ongoing recombination between the sex chromosomes.

On closer inspection, gene conversion events identified in this study span several exons that in turn are separated by extremely dissimilar intronic sequences. As GENECONV identifies potential gene conversion sites as long fragments of sequence bounded by variable sites, it estimates the maximum sequence length over which recombination acts. To avoid falsely identifying gene conversion sites, we excluded exons where GENECONV fragments span less than 50 bp. We used informative polymorphic sites and maximum-likelihood exon trees to establish the direction of gene conversion.

### SEQUENCE EVOLUTION OF Z- AND W-LINKED GENES

To assess the selective regime acting on Z and W coding sequences, we used the branch models and branch-site test in the CODEML package in PAML version 4.7 (Yang 2007).

Branch models (model = 2, nssites = 0) allow ω (*d*_N_/*d*_S_) to vary across branches. By comparing a model where ω is estimated to one where it is fixed to equal 1 (the expected *d*_N_/*d*_S_ under neutral evolution) or 0 (strict purifying selection) it is possible to assess the contribution of neutral and purifying selection to coding sequence evolution. To do this, we used the multiple sequence alignments described above (used to build gene trees and test for gene conversion). If *A. platyrhynchos* and *M. gallopavo* gametologs ceased recombining before the last common ancestor of the Galloanserae the following gene tree was specified (*T. guttata Z*, (((*G. gallus* W, *M. gallopavo* W), *A. platyrhynchos* W), ((*G. gallus* Z, *M. gallopavo* Z), *A. platyrhynchos* Z))) and if *A. platyrhynchos* gametologs diverged independently the following gene tree was used (*T. guttata Z*, (((*G. gallus* W, *M. gallopavo* W), (*G. gallus* Z, *M. gallopavo* Z)), (*A. platyrhynchos Z, A. platyrhynchos W*))).

The branch-site model (model = 2, nssites = 2) allows ω to vary across branches and among sites and permits tests to detect positive selection acting on a subset of sites along specific lineages. Under this model, we estimated ω across W and Z branches separately and then tested whether ω was significantly different to 1 using the settings fix_omega = 1, omega = 1.

## Results

### CHARACTERISING THE CODING CONTENT OF THE AVIAN W CHROMOSOME ACROSS SPECIES

We used a combination of bioinformatics, molecular genetic, and phylogenetic methods to validate putative W-linked genes and identify previously unknown W-linked coding content in the wild turkey (*M. gallopavo*) and mallard duck (*A. platyrhynchos*). Our combined dataset comprised 14 W-linked genes in *M. gallopavo* and 21 in *A. platyrhynchos*, together with 27 previously identified W-linked genes in the red jungle fowl (*G. gallus*) (Table1). Some W-linked genes are present in multiple copy number (Backstrom et al. 2005; Moghadam et al. 2012) and these W-linked genes correspond to seven gametologs in *M. gallopavo*, 11 gametologs in *A. platyrhynchos*, and 20 gametologs in *G. gallus*. To date, this is the largest cross-species dataset of W-linked coding sequences yet assembled.

**Table 1:** Known and newly identified *G. gallus*, *A. platyrhynchos*, and *M. gallopavo* W-linked coding genes

Name	Z-linked gene ID^1^	W-linked gene ID^1^
*G. gallus*		
*HINT1*	00428	22674,^2^ 22683,^3^ 22690,^3^ 22679,^3^
*CHD1*	14642	*CHDW*^3^
*RASA1*	17706	22611^2^,^3^
*KCMF1*	15391	14441
*MIER3*	14721	00140,^2^,^3^ 14584^2^,^3^
*SPIN*	14916	*SPINW*^3^
*HNRNPK*	12591	14366^2^,^3^
*CZH18ORF25*	01763	01585^2^,^3^
*VCP*	01986	00386^2^
*SIAT8C*	03049	26991
*ZFR*	03235	14545^2^,^3^
*NIPBL*	03605	13312^2^,^3^
*UBAP2*	13809	05785
*ATP5A1*	14644	01756^2^,^3^
*MADH2*	14697	14184
*BTF3*	13512	00395^2^,^3^
*UBE2R2*	01668	09227
*ZSWIM6*	14734	27170
*ZNF532*	02852	14003^3^
*RPL17L*	02696	22174^2^,^3^
*Novel*	-	29099
*Novel*	-	25736
*Novel*	-	25865
*M. gallopavo*		
*HINT1*	06655	16898, 09917, *HINTW*(AY713488.1)^4^
*RASA1*	08292	*RASA1W*(AH015047.1)^5^
*SPIN*	02500	*SPINW*(AH015050.1),^5^ 06743
*VCP*	-6	09037
*NIPBL*	01989	13606, *IDN3W*(AH015048.1)^5^
*UBAP2*	01837	*UBAP2W*(AY188758.1),^5^ 05595
*ATP5A1*	01414	09963
*MADH2*	*MADH2Z*^5^	*MADH2W*(AH015049.1)^5^
*Novel*	-	16394
*A. platyrhynchos*		
*CHD1*	09965	05191, 02506
*RASA1*	05627	05611, 11371, 10611
*KCMF1*	13426	03026, 03106
*MIER3*	06634	10850
*SPIN*	13922	02923
*HNRNPK*	10856	10986
*VCP*	06634	05806
*ZFR*	15627	15519
*NIPBL*	08473	10290, 05315, 10560, 02953, 03022
*UBE2R2*	03800	16000
*ZSWIM6*	06992	13555, 14338
*UBAP2*	06449	16155

1 Ensgalg000000(*G. gallus*)/Ensmgag000000(*M. gallopavo*)/Ensaplg000000(*A. platyrhynchos*).

2 Moghadam et al. (2012).

3 Ayers et al. (2013).

4 Backstrom et al. (2005).

5 Berlin and Ellegren (2006).

6 Amalgamation of Z and W sequences in current genome assembly.

### FOUR Z CHROMOSOME STRATA ARE CONSERVED IN THE GALLIFORMES

Sex chromosome strata are identified from clusters of gametologs with similar synonymous (*d*_S_) divergence estimates (Lahn and Page 1999; Skaletsky et al. 2003), which provide a measure of the relative length of time gametologs have been isolated from each other. Previously, we identified four evolutionary strata on the *G. gallus* Z chromosome, where recombination was suppressed progressively over 130 million years (Wright et al. 2012).

We used seven gametologs in *M. gallopavo* to independently verify the *G. gallus* strata. Z-linked physical position was conserved between *G. gallus* and *M. gallopavo* orthologs, and each stratum is represented by at least one *M. gallopavo* gametolog. For all gametologs, *d*_S_ estimates were consistent with orthologous *G. gallus* Z–W *d*_S_ estimates and 95% confidence intervals overlapped (Fig.1A, Table S2). Maximum-likelihood gene trees of *G. gallus* and *M. gallopavo* gametologs, with the zebra finch (*T. guttata*) Z-linked ortholog included as an outgroup, indicate that all four strata predate the *G. gallus*–*M. gallopavo* divergence estimated at roughly 30 MYA (Dimcheff et al. 2002a). For all seven gametologs, the gene trees clustered by sex chromosome rather than by species, and the phylogenetic grouping of *G. gallus* and *M. gallopavo* W-linked genes showed high bootstrap support (in all cases ≥95%, Figs.1A, S2), indicating that gametolog divergence predates the common ancestor of *G. gallus* and *M. gallopavo*. This provides independent verification that the four recombination suppression events previously identified in *G. gallus* are conserved across the Galliformes.

Divergence dates for recombination suppression in the youngest strata should predate the divergence between *G. gallus* and *M. gallopavo* approximately 30 Mya (Dimcheff et al. 2002a). Using a molecular clock that accounts for sex-specific mutation rates in Z- and W-linked genes (Wright et al. 2012), we found that Stratum III, corresponding to 17–43 Mb (*d*_S_ = 0.156–0.268), arose between 41 and 71 Mya and Stratum IV, corresponding to 0–11 Mb (*d*_S_ = 0.137–0.257), between 36 and 68 Mya (Fig.1A). These dates are consistent with the finding that four strata are conserved in the Galliformes.

### RECOMBINATION SUPPRESSION EVOLVED INDEPENDENTLY IN THE ANSERIFORMES

Using 11 newly identified *A. platyrhynchos* gametologs, we calculated *d*_S_ estimates and maximum-likelihood gene trees to assess the dynamics of recombination suppression and sex chromosome divergence. Avian genomes are relatively stable, with few major genomic rearrangements, potentially due to a lack of active transposons (Toups et al. 2011). As synteny of the Z chromosome is highly conserved among all extant birds (Vicoso et al. 2013b), including the Galloanserae *A. platyrhynchos* and *G. gallus* (Skinner et al. 2009), we took physical position of the *A. platyrhynchos* gametologs from the location of the corresponding *G. gallus* Z-linked ortholog. To ensure W-linked paralogs were not misidentified as Z-linked orthologs, we used PCR to confirm they were not physically female-limited.

For the three *A. platyrhynchos* gametologs located in the two oldest Galliform strata, *d*_S_ estimates were consistent with orthologous *G. gallus* Z–W *d*_S_ estimates, where 95% confidence intervals overlapped (Fig.1B, Table S3). Maximum-likelihood gene trees for all loci cluster by sex chromosome rather than by species, with 100% bootstrap support for the grouping of *G. gallus* and *A. platyrhynchos* W-linked orthologs distinct from Z-linked genes (Figs.1B, S3). For *RASA1Z* and *RASA1W*, we have coding sequence for *G. gallus*, *A. platyrhynchos*, and *M. gallopavo* gametologs, and W-linked sequences cluster together with 100% bootstrap support. The three gametolog sets therefore convergently indicate that the two oldest strata are conserved across the Galloanserae. These strata correspond to 45–51 Mb (*d*_S_ = 0.285–0.404, Conserved Stratum I), 54–61 Mb (*d*_S_ = 0.171–0.271, Conserved Stratum II), and arose between 75 and 106 Mya, and 45 and 71 Mya, respectively.

In contrast, of the eight *A. platyrhynchos* gametologs located in the youngest Galliform strata, *d*_S_ estimates for five gametologs are significantly lower than the corresponding *G. gallus* gametolog *d*_S_ estimates, where 95% confidence intervals are nonoverlapping (Fig.1B, Table S3). Of these eight *A. platyrhynchos* gametologs, four cluster together in maximum-likelihood gene trees with bootstrap values ≥95, indicating that recombination suppression occurred relatively recently and independently in the Anseriformes in this region. However, owing to low bootstrap values we were unable to resolve the phylogenetic topology for the other four gametologs. Using a molecular clock, we calculated that recombination ceased independently in Anseriformes between 0 and 39 Mya (Fig.1B), well after the divergence of Galliformes and Anseriformes approximately 90 Mya (van Tuinen and Hedges 2001).

### HETEROGENEOUS DIVERGENCE OF YOUNG ANSERIFORM GAMETOLOGS

There is marked heterogeneity in *d*_S_ across the youngest region of the Anseriform sex chromosomes, ranging from 0 to 0.148. This variation is greater than that observed across any other avian strata, and is not a result of a single outlier. Three gametologs have *d*_S_ < 0.02, indicating either very recent divergence or persistent recombination. We verified that Z- and W-linked coding sequences were distinct using female-limited SNPs identified from RNA-seq data and W-linked SNPs from Ensembl annotated sequences. Therefore, the heterogeneity in divergence is not consistent with instantaneous suppression of recombination resulting from a large-scale intra-chromosomal event such as an inversion. Instead, this may suggest more gradual divergence of gametologs with residual recombination across large tracts of the sex chromosomes.

To further assess the possibility of ongoing recombination between gametologs, we identified signatures of gene conversion between Z- and W-linked coding sequences in *G. gallus*, *A. platyrhynchos*, and *M. gallopavo* using GENECONV (Sawyer 1999). Three gametologs in *A. platyrhynchos* showed evidence of significant inner gene conversion following a permutation test with 10,000 iterations (Table2). We repeated the analysis allowing mismatches between gene conversion fragments (*gscale* = 2) and found no significant difference between the results. These gametologs are uniformly distributed across the Z chromosome, located in Conserved Strata I, II, and Anseriform-specific Stratum III, indicating ongoing recombination along the whole length of the avian sex chromosomes. In total, we detected four Z- and four W-linked exons subject to gene conversion (Table2). Of these, we could determine the direction of gene conversion for the exons in the Conserved Strata I and II. Informative variant sites indicate genetic material was transferred from *KCMF1W* to *KCMF1Z* and from *CHD1Z* to *CHD1W*. Exon tree topologies support the proposed direction of gene conversion in *KCMF1* and cluster by species for *CHD1*, revealing putative gene conversion across *G. gallus CHD1* gametologs, the signal of which was not detected with GENECONV.

**Table 2:** Gene conversion between gametologs in *A. platyrhynchos*

	Gene			Spanning Z-linked	Spanning W-linked	Length of gene
Gene name	ID (Z/W)^a^	Stratum	*P*-value b^/^c	exon no.^d^	exon no.^d^	conversion track (bp)
*CHD1*	09965/	Conserved I	0.0006/	27	7	174
	05191		0.0027*			
*KCMF1*	13426/	Conserved II	0.0056/	4	3	142
	03026		0.0273			
*HNRNPK*	10856/	Anseriform-specific	0.0081/	10	8	255
	10986	III	0.0294			
*HNRNPK*	10856/	Anseriform-specific	0.0314/	18	13	159
	10986	III	0.0775			

a Ensaplg000000.

b Simulated *P*-values are based on 10,000 permutations.

c Karlin–Altshul *P*-values are Bonferroni-corrected KA (BLAST-like).

d Exons where gene conversion fragments span <50 bp are not listed.

* Significant after correcting for multiple comparisons.

Gene conversion between gametologs can lower *d*_S_ estimates and generate molecular gene trees where gametologs cluster by species. This may lead to false conclusions that recombination was suppressed independently in a given lineage. We detected no gene conversion between the four *A. platyrhynchos* gametologs where gene trees cluster by species, or the three gametologs with *d*_S_ < 0.02. However, previous studies have shown that GENECONV's power to detect gene conversion events between similar sequences is limited (McGrath et al. 2009; Ezawa et al. 2010).

We recalculated *d*_S_ for three *A. platyrhynchos* gametologs after removing exons subject to gene conversion and as expected, the estimated divergence time increased. However, the range of divergence dates over which recombination was suppressed in Conserved Stratum I and II remained constant. The recalculated gene tree for the gametolog in Anseriform-specific Stratum III revealed a complex mosaic of recombination across specific exons. Before excluding the exons subject to gene conversion, the phylogenetic topology was unresolvable, however after removal of these exons, *A. platyrhynchos* and *G. gallus HNRNPKW* clustered together with high bootstrap support (≥95%).

### COMPARATIVE ANALYSIS OF W-LINKED CODING CONTENT EVOLUTION

Studies examining the evolutionary forces acting after recombination suppression are mainly limited to male heterogametic systems (Chibalina and Filatov 2011; Hughes et al. 2012; Bachtrog 2013). As a result, the factors driving the differentiation and degeneration of Z and W sex chromosomes are partially unclear. Now equipped with the largest dataset of W-linked genes to date, we used the branch models and branch-site test in the CODEML package in PAML version 4.7 (Yang 2007) to assess the selective regime driving Z and W coding sequence change over 90 million years of avian evolution.

For W-linked branches, ω (*d*_N_/*d*_S_) was significantly lower than 1 in 14 gametolog families, indicating these genes are evolving primarily due to purifying selection (Tables3, S4). The exception was *HINT1W*, where ω was not significantly different to 1. However, *HINT1W* is a known ampliconic gene with documented gene conversion among W-linked copies (Backstrom et al. 2005). This likely violates the assumptions made in CODEML, where *d*_S_ is equivalent to the neutral mutation rate (Yang 2007). For Z-linked branches, ω was not significantly different to 0 across four loci, indicating these are extremely conserved and evolving under strict purifying selection. For the other 10 loci, ω was significantly different to both 1 and 0 for all but one locus, *UBAP2Z* (Table S5).

**Table 3:** Branch models and branch-site test for W-linked branches

Gene	Species		Branch model test	Branch model test	Branch-site test
name	included^1^	ω	(ω=1) likelihood ratio	(ω=0) likelihood ratio	(ω =1) likelihood ratio
*HINT1W*	G, M, T	1.10	0.05	3697.05**	0.05
*CHD1W*	G, A, T	0.14	94.45**	7012.15**	0.00
*RASA1W*	G, A, M, T	0.09	31.33**	1386.95**	0.00
*KCMF1W*	G, A, T	0.22	35.49**	5674.71**	0.22
*MIER3W*	G, A, T	0.24	17.97**	167.25**	0.00
*SPINW*	G, A, M, T	0.09	28.45**	1749.98**	0.00
*HNRNPKW*	G, A, T	0.03	108.75**	84.21**	0.03
*VCPW*	G, A, T	0.03	25.58**	19.13**	0.16
*ZFRW*	G, A, T	0.18	27.16**	173.50**	0.00
*NIPBLW*	G, A, T	0.05	44.08**	63.91**	0.00
*NIPBLW*	G, M, T	0.03	38.40**	343.5**	0.00
*UBAP2W*	G, M, T	0.15	6.19*	354.82**	0.00
*ATP5A1W*	G, M, T	0.09	16.54**	650.83**	3.58*
*MADH2W*	G, M, T	0.06	22.37**	333.50**	0.01
*UBE2R2W*	G, A, T	0.04	15.03**	17.39**	0.00
*ZSWIM6W*	G, A, T	0.25	11.25**	128.88**	0.00

1 G = *G. gallus*, M = *M. gallopavo*, A = *A. platyrhynchos*, T = *T. guttata*.

** *P*-value < 0.01.

* *P*-value < 0.05.

Likelihood values for each model and the gene IDs are listed in Table S4. Significant likelihood ratios are in bold.

We tested for positive selection using the branch-site model. This model allows ω to vary across both branches and among sites, and tests for adaptive evolution acting on a subset of codons along a branch(es) defined a priori (Zhang et al. 2005). We found no evidence of positive selection on any Z-linked gene families (Table S5) but found significant evidence for positive selection acting on one W-linked loci, *ATP5A1W* (proportion of sites where ω > 1 was 0.012 and ω = 17.70) (Tables3, S4).

Gene conversion violates the assumption in PAML that *d*_S_ is constant across a gene and the GC bias in the mismatch repair process can inflate ω (*d*_N_/*d*_S_) (Berglund et al. 2009). Consequentially, previous studies have found that gene conversion can lead to false signals of positive selection (Casola and Hahn 2009; Ratnakumar et al. 2010). Although we found no evidence of gene conversion between *ATP5A1W* and *ATP5A1Z* in any species, *ATP5A1W* is present in two copies in the *G. gallus* genome (Moghadam et al. 2012) and may be subject to intra-chromosomal gene conversion. In contrast, our polymorphism data indicate there is only a single copy in the *M. gallopavo* genome, as we failed to identify polymorphisms in this locus within any single female, which would indicate multiple copy number. To exclude the possibility that gene conversion between W-linked *G. gallus* paralogs is driving a false signal of positive selection, we removed *G. gallus ATP5A1W* and repeated the analysis. After excluding this gene, the branch-site test for positive selection remained significant (proportion of sites where ω > 1 was 0.006 and ω = 41.87). *ATP5A1W* encodes a mitochondrial ATP synthase subunit. As the W chromosome and mitochondrial genome are both maternally inherited and effectively linked (Berlin et al. 2007), one plausible explanation for this signature of positive evolution may be that it reflects adaptive coevolution.

## Discussion

The process of recombination suppression is a crucial component of sex chromosome evolution, facilitating the divergence and differentiation of gametologs (Charlesworth and Charlesworth 1978). Studies from highly differentiated sex chromosomes suggest that recombination is suppressed instantaneously in a stepwise manner by intra-chromosomal rearrangements (Lahn and Page 1999; Pandey et al. 2013). However, our ability to determine whether inversions are a cause or consequence of this process is hampered by the limited numbers of sex-linked genes in these species. Conversely, in newly evolved sex chromosomes, recombination appears to be suppressed in a gradual and heterogeneous nature (Chibalina and Filatov 2011; Muyle et al. 2012; Bergero et al. 2013; Natri et al. 2013; Qiu et al. 2013).

We used a comparative approach to study the dynamics of sex chromosome recombination suppression on the largest collection of W-linked coding content yet assembled across 90 million years of avian evolutionary history. Our results reveal that recombination between the Z and W chromosomes ceased independently multiple times across different avian lineages. The pattern we uncover is consistent with both the inversion model (Charlesworth et al. 2005) where evolutionary strata form in a stepwise process, and heterogeneous and locus specific recombination suppression over short-term periods. Additionally, we show that recombination suppression is not necessarily complete, as highly diverged gametologs recombine across specific exons via gene conversion.

### DYNAMICS OF SEX CHROMOSOME DIVERGENCE

**Figure 2:**
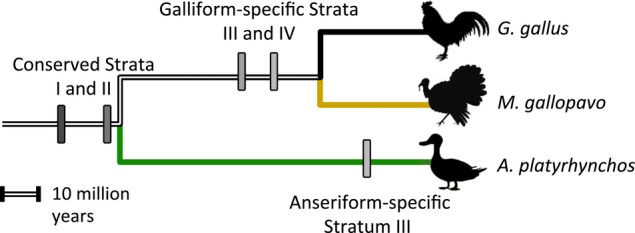
Independent expansion of the nonrecombining regions across the Galloanserae. Phylogeny illustrating the independent cessation of recombination on the sex chromosomes in the Galliformes and Anseriformes. Estimated date of recombination suppression (millions of years), calculated using a molecular clock that accounts for sex-specific mutation rates in Z- and W-linked genes, is mapped onto the phylogeny.

Using both maximum-likelihood gene trees and synonymous divergence estimates, we confirm that four strata are conserved across the Galliformes (Fig.2). These four strata were previously identified in *G. gallus* (Wright et al. 2012), and follow the same stepwise pattern observed across the mammalian X chromosome (Lahn and Page 1999; Sandstedt and Tucker 2004; Pearks Wilkerson et al. 2008; Van Laere et al. 2008). Using newly identified gametologs, we were able to refine the dates at which recombination was suppressed across the Galliform strata. We find that the oldest strata originated approximately 90 Mya, and the youngest 46 Mya, both of which are consistent with the divergence date between *G. gallus* and *M. gallopavo* (Dimcheff et al. 2002a).

Similarly, maximum-likelihood gene trees reveal that the two oldest Galliform strata are conserved on the Anseriform Z chromosome. In contrast, recombination suppression is heterogeneous across the remainder of the *A. platyrhynchos* sex chromosomes, and the topology of gene trees revealed that gametologs diverged independently in this region (Fig.2). Divergence estimates vary from 0 to 39 Mya, a range that is greater than that observed across any other avian strata, and not a result of a single outlier. We verified that Z- and W-linked sequences were in fact distinct using polymorphism data and Ensembl coding sequences. This heterogeneity in divergence is not consistent with instantaneous suppression of recombination resulting from a large-scale intra-chromosomal event and is instead indicative of Z–W divergence combined with ongoing recombination. However, due to the limited number of gametologs, we lack sufficient power to formally test for differences in the variance of *d*_S_ across strata.

The lack of positional information for the *A. platyrhynchos* Z chromosome means we cannot rule out the possibility that Anseriform-specific Stratum III is actually made up of two distinct strata formed by a lineage specific inversion. Under this scenario, the three gametologs with *d*_S_ estimates <0.02 would comprise the youngest stratum with ongoing recombination between the W and Z orthologs. Positional information may support this scenario, as two of the three gametologs are located between 0 and 10 Mb on the Z chromosome.

Some gametologs are subject to gene conversion, lending further support to the notion that recombination suppression is not complete. Three *A. platyrhynchos* gametologs showed evidence of significant gene conversion, and were distributed across the length of the Z chromosome. Therefore, even for highly differentiated gametologs, certain exons are prevented from diverging. This is consistent with previous studies documenting gene conversion between X- and Y-linked orthologs in mammals (Iwase et al. 2010; Trombetta et al. 2014). Gene conversion may therefore be an important mechanism halting the degeneration of sex-limited gametologs (Rosser et al. 2009) in addition to the intra-chromosomal gene conversion documented on the avian W and mammalian Y chromosomes (Backstrom et al. 2005; Marais et al. 2010; Hallast et al. 2013; Bellott et al. 2014). Interestingly, a previous study used whole chromosome painting to show that the *A. platyrhynchos* W chromosome is highly divergent in comparison to closely related avian W chromosomes (Stiglec et al. 2007). We find a high degree of conservation among coding genes, indicating exon-specific gene conversion may facilitate large-scale turnover of intronic and repetitive regions while still conserving gene function.

This heterogeneous pattern of sex chromosome divergence and ongoing recombination is consistent with *G. aculeatus* and *S. latifolia* sex chromosomes. The *S. latifolia* XY system originated 10 Mya, and a neo-sex chromosome formed 2 Mya in *G. aculeatus*. In both these system, recombination suppression evolves heterogeneously (Chibalina and Filatov 2011; Muyle et al. 2012; Bergero et al. 2013; Natri et al. 2013; Qiu et al. 2013; Roesti et al. 2013). The underlying mechanism of recombination suppression is unclear, but changes in heterochromatin and methylation, which have been shown to alter patterns of recombination (Mirouze et al. 2012), or local changes in the binding sequence that recruits recombination machinery to the chromosome (Baudat et al. 2010; Myers et al. 2010), may be responsible.

Our divergence estimates for recombination suppression are consistent with divergence dates for *G. gallus*, *A. platyrhynchos*, and *M. gallopavo* with the exception of Conserved Stratum II. Using a molecular clock that accounts for sex-specific mutation rates in Z- and W-linked genes, the divergence of Conserved Stratum II was estimated to have occurred between 45 and 71 Mya. Galliformes and Anseriformes diverged 90 Mya (van Tuinen and Hedges 2001). Although these estimates are not vastly distant, they are inconsistent and may indicate that the molecular clock underestimates the time of gametolog divergence due to the unique evolutionary forces acting on sex chromosomes (Bachtrog et al. 2011).

### EVOLUTION OF HETEROGAMETIC SEX CHROMOSOMES AFTER RECOMBINATION SUPPRESSION

Studies examining the evolutionary forces acting after recombination has ceased have mainly been limited to male heterogametic systems, such as the Y chromosomes of mammals and *Drosophila* (Chibalina and Filatov 2011; Hughes et al. 2012; Bachtrog 2013). These studies have uncovered strong purifying selection acting on the Y chromosome (Kaiser et al. 2011; Hughes et al. 2012; Bellott et al. 2014; Wilson Sayres et al. 2014) together with signatures of positive selection (Gerrard and Filatov 2005; Li et al. 2013). However, the selective forces driving the evolution of the W chromosome are unclear. This is partly due to a previous paucity of known W-linked genes, which has restricted the potential of interspecific analyses (Berlin and Ellegren 2006).

Theoretical models predict key differences between W and Y chromosome evolution (Kirkpatrick and Hall 2004; Mank 2012; Wright and Mank 2013). When sexual selection is acting primarily on males, the Y chromosome has a lower effective population size than W-linked sequences (Wright and Mank 2013), and is subject to male-mutation bias (Kirkpatrick and Hall 2004). These factors have been hypothesized to result in rapid degeneration of Y-linked coding sequences, a pattern that has been observed on *Drosophila* and primate Y chromosomes (Hughes et al. 2010, 2012; Bachtrog 2013).

Using the largest catalog of W-linked genes to date, we find that avian W-linked genes are evolving with a significant contribution of purifying selection. This is consistent with a previous study showing that sex-specific selection drives evolutionary changes in the expression of W-linked genes (Moghadam et al. 2012), and indicates that although the W chromosome does not determine sex directly (Smith et al. 2009), it has an important role in encoding sex-specific fitness. The large number of genes conserved across 90 million years of avian evolution is also potentially indicative of lower degeneration rates on the W compared to the Y chromosome.

We found evidence of positive selection in one W-linked gene that encodes a mitochondrial ATP synthase subunit. As the W chromosome and mitochondria are both maternally inherited and are effectively linked (Berlin et al. 2007), we might expect strong coevolution between these portions of the genome, and this may be reflected by rapid divergence in sequence across different avian lineages.

Our findings indicate that adaptive evolution together with conservation of gene sequence is not restricted to male heterogametic systems and may be a fundamental feature of heterogametic sex chromosome evolution.

### CONCLUDING REMARKS

Recombination suppression is a key force driving sex chromosome evolution, and sex chromosomes are therefore important for the study of genome evolution, particularly the interplay between selective forces and recombination. Here, we use a comparative approach to examine short- and long-term dynamics of sex chromosome evolution. Our results reveal that recombination has ceased multiple and independent times over 90 million years of avian evolution. We show that sex chromosome divergence over the long term is characterized by regions of similar divergence among gametologs, consistent with the inversion model of sex chromosome evolution (Lahn and Page 1999; Charlesworth et al. 2005; Vicoso et al. 2013a). In contrast, over short time periods we observe heterogeneous and locus specific divergence. Additionally, we uncover gene conversion between highly diverged gametologs across both long and short evolutionary trajectories. Moreover, if sex chromosomes do evolve via inversions, additional mechanisms may be required to completely suppress recombination. Our data also show that the W chromosome is evolving primarily due to purifying selection, although we also detect the signature of positive selection at one locus. Our findings indicate that the W chromosome plays an important role in encoding sex-specific fitness, and potentially exhibits a lower rate of degeneration compared to Y chromosome.

## Supporting Information

Disclaimer: Supplementary materials have been peer-reviewed but not copyedited.

Figure S1 Identification of W-linked genes.

Figure S2 Gene trees for *M. gallopavo* gametologs.

Figure S3 Gene trees for *A. platyrhynchos* gametologs.

Table S1 Sequence divergence between *G. gallus* gametologs. **Table S2**. Sequence divergence between *M. gallopavo* gametologs. **Table S3**. Sequence divergence between *A. platyrhynchos* gametologs. **Table S4**. Branch models and branch-site test across W-linked branches. **Table S5**. Branch models and branch-site test across Z-linked branches.

## References

[b1] Altschul SF, Gish W, Miller W, Myers EW, Lipman DJ (1990). Basic local alignment search tool. J. Mol. Biol.

[b3] Axelsson E, Smith NGC, Sundström H, Berlin S, Ellegren H (2004). Male-biased mutation rate and divergence in autosomal, Z-linked and W-linked introns of chicken and turkey. Mol. Biol. Evol.

[b2] Axelsson E, Hultin-Rosenberg L, Brandström M, Zwahlen M, Clayton DF, Ellegren H (2008). Natural selection in avian protein-coding genes expressed in brain. Mol. Ecol.

[b4] Ayers KL, Davidson NM, Demiyah D, Roeszler KN, Gruetzner F, Sinclair AH, Oshlack A, Smith CA (2013). RNA sequencing reveals sexually dimorphic gene expression before gonadal differentiation in chicken and allows comprehensive annotation of the W-chromosome. Genome Biol.

[b5] Bachtrog D (2013). Y-chromosome evolution: emerging insights into processes of Y-chromosome degeneration. Nat. Rev. Genet.

[b6] Bachtrog D, Hom E, Wong KM, Maside X, de Jong P (2008). Genomic degradation of a young Y chromosome in *Drosophila miranda*. Genome Biol.

[b7] Bachtrog D, Kirkpatrick M, Mank JE, McDaniel SF, Pires JC, Rice WR, Valenzuela N (2011). Are all sex chromosomes created equal?. Trends Genet.

[b8] Backstrom N, Ceplitis H, Berlin S, Ellegren H (2005). Gene conversion drives the evolution of HINTW, an ampliconic gene on the female-specific avian W chromosome. Mol. Biol. Evol.

[b9] Baudat F, Buard J, Grey C, Fledel-Alon A, Ober C, Przeworski M, Coop G, de Massy B (2010). PRDM9 Is a major determinant of meiotic recombination hotspots in humans and mice. Science.

[b10] Bellott DW, Hughes JF, Skaletsky H, Brown LG, Pyntikova T, Cho T-J, Koutseva N, Zaghlul S, Graves T, Rock S (2014). Mammalian Y chromosomes retain widely expressed dosage-sensitive regulators. Nature.

[b11] Bergero R, Qiu S, Forrest A, Borthwick H, Charlesworth D (2013). Expansion of the pseudo-autosomal region and ongoing recombination suppression in the *Silene latifolia* sex chromosomes. Genetics.

[b12] Berglund J, Pollard KS, Webster MT (2009). Hotspots of biased nucleotide substitutions in human genes. PLoS Biol.

[b14] Berlin S, Ellegren H (2006). Fast accumulation of nonsynonymous mutations on the female-specific W chromosome in birds. J. Mol. Evol.

[b13] Berlin S, Brandstrom M, Backstrom N, Axelsson E, Smith N, Ellegren H (2006). Substitution rate heterogeneity and the male mutation bias. J. Mol. Evol.

[b15] Berlin S, Tomaras D, Charlesworth B (2007). Low mitochondrial variability in birds may indicate Hill-Robertson effects on the W chromosome. Heredity.

[b16] Busby M, Gray J, Costa A, Stewart C, Stromberg M, Barnett D, Chuang J, Springer M, Marth G (2011). Expression divergence measured by transcriptome sequencing of four yeast species. BMC Genomics.

[b17] Casola C, Hahn MW (2009). Gene conversion among paralogs results in moderate false detection of positive selection using likelihood methods. J. Mol. Evol.

[b18] Charlesworth B (1991). The evolution of sex chromosomes. Science.

[b19] Charlesworth B, Charlesworth D (1978). A model for the evolution of dioecy and gynodioecy. Am. Nat.

[b20] Charlesworth B, Charlesworth D (2000). The degeneration of Y chromosomes. Philos. Trans. R. Soc. Lond. Ser. B Biol. Sci.

[b21] Charlesworth D, Charlesworth B, Marais G (2005). Steps in the evolution of heteromorphic sex chromosomes. Heredity.

[b22] Chen N, Bellott DW, Page DC, Clark AG (2012). Identification of avian W-linked contigs by short-read sequencing. BMC Genomics.

[b23] Chibalina MV, Filatov DA (2011). Plant Y chromosome degeneration is retarded by haploid purifying selection. Curr. Biol.

[b24] Dalloul RA, Long JA, Zimin AV, Aslam L, Beal K, Blomberg LA, Bouffard P, Burt DW, Crasta O, Crooijmans RPMA (2010). Multi-platform next-generation sequencing of the domestic turkey (*Meleagris gallopavo*): genome assembly and analysis. PLoS Biol.

[b25] Dalloul RA, Zimin AV, Settlage RE, Kim S, Reed KM (2014). Next-generation sequencing strategies for characterizing the turkey genome. Poult. Sci.

[b26] Dimcheff DE, Drovetski SV, Mindell DP (2002a). Phylogeny of *Tetraoninae* and other galliform birds using mitochondrial 12S and ND2 genes. Mol. Phylogenet. Evol.

[b27] Dimcheff DE, Drovetski SV, Mindell DP (2002b). Phylogeny of *Tetraoninae* and other galliform birds using mitochondrial 12S and ND2 genes. Mol. Phylogenet. Evol.

[b28] Edgar RC (2004a). MUSCLE: a multiple sequence alignment method with reduced time and space complexity. BMC Bioinformatics.

[b29] Edgar RC (2004b). MUSCLE: multiple sequence alignment with high accuracy and high throughput. Nucleic Acids Res.

[b31] Ezawa K, Oota S, Saitou N (2006). Genome-wide search of gene conversions in duplicated genes of mouse and rat. Mol. Biol. Evol.

[b30] Ezawa K, Ikeo K, Gojobori T, Saitou N (2010). Evolutionary pattern of gene homogenization between primate-specific paralogs after human and macaque speciation using the 4–2–4 method. Mol. Biol. Evol.

[b32] Flicek P, Ahmed I, Amode MR, Barrell D, Beal K, Brent S, Carvalho-Silva D, Clapham P, Coates G, Fairley S (2013). Ensembl 2013. Nucleic Acids Res.

[b33] Gerrard DT, Filatov DA (2005). Positive and negative selection on mammalian Y chromosomes. Mol. Biol. Evol.

[b34] Graur D, Li W-H (2000). Fundamentals of molecular evolution.

[b35] Hallast P, Balaresque P, Bowden GR, Ballereau S, Jobling MA (2013). Recombination dynamics of a human Y-chromosomal palindrome: rapid GC-biased gene conversion, multi-kilobase conversion tracts, and rare inversions. PLoS Genet.

[b36] Harlid AB, Berlin S, Smith NGC, Mosller AP, Ellegren H (2003). Life history and the male mutation bias. Evolution.

[b37] Hedges SB (2002). The origin and evolution of model organisms. Nat. Rev. Genet.

[b38] Huang Y, Li Y, Burt DW, Chen H, Zhang Y, Qian W, Kim H, Gan S, Zhao Y, Li J (2013). The duck genome and transcriptome provide insight into an avian influenza virus reservoir species. Nat. Genet.

[b40] Hughes JF, Skaletsky H, Pyntikova T, Graves TA, van Daalen SKM, Minx PJ, Fulton RS, McGrath SD, Locke DP, Friedman C (2010). Chimpanzee and human Y chromosomes are remarkably divergent in structure and gene content. Nature.

[b39] Hughes JF, Skaletsky H, Brown LG, Pyntikova T, Graves T, Fulton RS, Dugan S, Ding Y, Buhay CJ, Kremitzki C (2012). Strict evolutionary conservation followed rapid gene loss on human and rhesus Y chromosomes. Nature.

[b41] Iwase M, Satta Y, Hirai H, Hirai Y, Takahata N (2010). Frequent gene conversion events between the X and Y homologous chromosomal regions in primates. BMC Evol. Biol.

[b42] Kaiser VB, Zhou Q, Bachtrog D (2011). Nonrandom gene loss from the *Drosophila miranda* neo-Y chromosome. Genome Biol. Evol.

[b43] Kim D, Pertea G, Trapnell C, Pimentel H, Kelley R, Salzberg SL (2013). TopHat2: accurate alignment of transcriptomes in the presence of insertions, deletions and gene fusions. Genome Biol.

[b44] Kirkpatrick M, Hall DW (2004). Male-biased mutation, sex linkage, and the rate of adaptive evolution. Evolution.

[b45] Lahn BT, Page DC (1999). Four evolutionary strata on the human X chromosome. Science.

[b46] Langmead B, Salzberg SL (2012). Fast gapped-read alignment with Bowtie 2. Nat. Methods.

[b47] Langmead B, Trapnell C, Pop M, Salzberg SL (2009). Ultrafast and memory-efficient alignment of short DNA sequences to the human genome. Genome Biol.

[b48] Lemaitre C, Braga MDV, Gautier C, Sagot M-F, Tannier E, Marais GAB (2009). Footprints of inversions at present and past pseudoautosomal boundaries in human sex chromosomes. Genome Biol. Evol.

[b49] Li G, Davis BW, Raudsepp T, Wilkerson AJP, Mason VC, Ferguson-Smith M, O'Brien PC, Waters PD, Murphy WJ (2013). Comparative analysis of mammalian Y chromosomes illuminates ancestral structure and lineage-specific evolution. Genome Res.

[b50] Lohse M, Bolger AM, Nagel A, Fernie AR, Lunn JE, Stitt M, Usadel B (2012). RobiNA: a user-friendly, integrated software solution for RNA-Seq-based transcriptomics. Nucleic Acids Res.

[b51] Mank JE (2012). Small but mighty: the evolutionary dynamics of W and Y sex chromosomes. Chromosome Res.

[b52] Marais GAB, Campos PRA, Gordo I (2010). Can intra-Y gene conversion oppose the degeneration of the human Y chromosome? A simulation study. Genome Biol. Evol.

[b53] McGrath CL, Casola C, Hahn MW (2009). Minimal effect of ectopic gene conversion among recent duplicates in four mammalian genomes. Genetics.

[b54] Mirouze M, Lieberman-Lazarovich M, Aversano R, Bucher E, Nicolet J, Reinders J, Paszkowski J (2012). Loss of DNA methylation affects the recombination landscape in *Arabidopsis*. Proc. Natl. Acad. Sci. USA.

[b55] Moghadam HK, Pointer MA, Wright AE, Berlin S, Mank JE (2012). W chromosome expression responds to female-specific selection. Proc. Natl. Acad. Sci. USA.

[b56] Muyle A, Zemp N, Deschamps C, Mousset S, Widmer A, Marais GAB (2012). Rapid de novo evolution of X chromosome dosage compensation in *Silene latifolia*, a plant with young sex chromosomes. PLoS Biol.

[b57] Myers S, Bowden R, Tumian A, Bontrop RE, Freeman C, MacFie TS, McVean G, Donnelly P (2010). Drive against hotspot motifs in primates implicates the PRDM9 gene in meiotic recombination. Science.

[b58] Nam K, Ellegren H (2008). The chicken (*Gallus gallus*) Z chromosome contains at least three nonlinear evolutionary strata. Genetics.

[b59] Natri HM, Shikano T, Merila J (2013). Progressive recombination suppression and differentiation in recently evolved neo-sex chromosomes. Mol. Biol. Evol.

[b60] Ohno S (1967). Sex chromosomes and sex linked genes.

[b61] Pandey RS, Wilson Sayres MA, Azad RK (2013). Detecting evolutionary strata on the human X chromosome in the absence of gametologous Y-linked sequences. Genome Biol. Evol.

[b62] Pearks Wilkerson AJ, Raudsepp T, Graves T, Albracht D, Warren W, Chowdhary BP, Skow LC, Murphy WJ (2008). Gene discovery and comparative analysis of X-degenerate genes from the domestic cat Y chromosome. Genomics.

[b63] Qiu S, Bergero R, Charlesworth D (2013). Testing for the footprint of sexually antagonistic polymorphisms in the pseudoautosomal region of a plant sex chromosome pair. Genetics.

[b64] Ratnakumar A, Mousset S, Glémin S, Berglund J, Galtier N, Duret L, Webster MT (2010). Detecting positive selection within genomes: the problem of biased gene conversion. Philos. Trans. R. Soc. Lond. Ser. B Biol. Sci.

[b76] R Core Team (2011). R: a language and environment for statistical computing. R Foundation for Statistical Computing, Vienna, Austria. http://www.R-project.org.

[b96] Roesti M, Moser D, Berner D (2013). Recombination in the threespine stickleback genome–patterns and consequences. Mol. Ecol.

[b65] Ross MT, Grafham DV, Coffey AJ, Scherer S, McLay K, Muzny D, Platzer M, Howell GR, Burrows C, Bird CP (2005). The DNA sequence of the human X chromosome. Nature.

[b66] Rosser ZH, Balaresque P, Jobling MA (2009). Gene conversion between the X chromosome and the male-specific region of the Y chromosome at a translocation hotspot. Am. J. Med. Genet.

[b67] Sandstedt SA, Tucker PK (2004). Evolutionary strata on the mouse X chromosome correspond to strata on the human X chromosome. Genome Res.

[b68] Sawyer SA (1999). GENECONV: a computer package for the statistical detection of gene conversion. www.math.wustl.edu/~sawyer/geneconv/.

[b69] Schemper M (2003). Predictive accuracy and explained variation. Stat. Med.

[b70] Skaletsky H, Kuroda-Kawaguchi T, Minx PJ, Cordum HS, Hillier L, Brown LG, Repping S, Pyntikova T, Ali J, Bieri T (2003). The male-specific region of the human Y chromosome is a mosaic of discrete sequence classes. Nature.

[b71] Skinner BM, Robertson LBW, Tempest HG, Langley EJ, Ioannou D, Fowler KE, Crooijmans RPMA, Hall AD, Griffin DK, Voelker M (2009). Comparative genomics in chicken and Pekin duck using FISH mapping and microarray analysis. BMC Genomics.

[b72] Smith CA, Roeszler KN, Ohnesorg T, Cummins DM, Farlie PG, Doran TJ, Sinclair AH (2009). The avian Z-linked gene DMRT1 is required for male sex determination in the chicken. Nature.

[b73] Stiglec R, Ezaz T, Graves JAM (2007). A new look at the evolution of avian sex chromosomes. Cytogenet. Genome Res.

[b74] Suh A, Kriegs JO, Brosius J, Schmitz J (2011). Retroposon insertions and the chronology of avian sex chromosome evolution. Mol. Biol. Evol.

[b75] Tamura K, Peterson D, Peterson N, Stecher G, Nei M, Kumar S (2011). MEGA5: molecular evolutionary genetics analysis using maximum likelihood, evolutionary distance, and maximum parsimony methods. Mol. Biol. Evol.

[b77] Tian L, Cai T, Goetghebeur E, Wei LJ (2007). Model evaluation based on the sampling distribution of estimated absolute prediction error. Biometrika.

[b78] Toups MA, Pease JB, Hahn MW (2011). No excess gene movement is detected off the avian or lepidopteran Z chromosome. Genome Biol. Evol.

[b80] Trapnell C, Pachter L, Salzberg SL (2009). TopHat: discovering splice junctions with RNA-Seq. Bioinformatics.

[b81] Trapnell C, Williams BA, Pertea G, Mortazavi A, Kwan G, van Baren MJ, Salzberg SL, Wold BJ, Pachter L (2010). Transcript assembly and quantification by RNA-Seq reveals unannotated transcripts and isoform switching during cell differentiation. Nat. Biotechnol.

[b79] Trapnell C, Hendrickson DG, Sauvageau M, Goff L, Rinn JL, Pachter L (2013). Differential analysis of gene regulation at transcript resolution with RNA-seq. Nat. Biotechnol.

[b82] Trombetta B, Sellitto D, Scozzari R, Cruciani F (2014). Inter- and intra-species phylogenetic analyses reveal extensive X-Y gene conversion in the evolution of gametologous sequences of human sex chromosomes. Mol. Biol. Evol.

[b83] Tsuda Y, Nishida-Umehara C, Ishijima J, Yamada K, Matsuda Y (2007). Comparison of the Z and W sex chromosomal architectures in elegant crested tinamou (*Eudromia elegans*) and ostrich (*Struthio camelus*) and the process of sex chromosome differentiation in palaeognathous birds. Chromosoma.

[b84] Van Laere A-S, Coppieters W, Georges M (2008). Characterization of the bovine pseudoautosomal boundary: documenting the evolutionary history of mammalian sex chromosomes. Genome Res.

[b85] van Tuinen M, Hedges SB (2001). Calibration of avian molecular clocks. Mol. Biol. Evol.

[b86] Vicoso B, Emerson JJ, Zektser Y, Mahajan S, Bachtrog D (2013a). Comparative sex chromosome genomics in snakes: differentiation, evolutionarystrata, and lack of global dosage compensation. PLoS Biol.

[b87] Vicoso B, Kaiser VB, Bachtrog D (2013b). Sex-biased gene expression at homomorphic sex chromosomes in emus and its implication for sex chromosome evolution. Proc. Natl. Acad. Sci. USA.

[b88] Webster MT, Axelsson E, Ellegren H (2006). Strong regional biases in nucleotide substitution in the chicken genome. Mol. Biol. Evol.

[b89] Wilson MA, Makova KD (2009). Evolution and survival on eutherian sex chromosomes. PLoS Genet.

[b90] Wilson Sayres MA, Lohmueller KE, Nielsen R (2014). Natural selection reduced diversity on Human Y chromosomes. PLoS Genet.

[b91] Wright AE, Mank JE (2013). The scope and strength of sex-specific selection in genome evolution. J. Evol. Biol.

[b92] Wright AE, Moghadam HK, Mank JE (2012). Trade-off between selection for dosage compensation and masculinization on the avian Z chromosome. Genetics.

[b93] Yang Z (2007). PAML 4: phylogenetic analysis by maximum likelihood. Mol. Biol. Evol.

[b94] Zhang J, Nielsen R, Yang Z (2005). Evaluation of an improved branch-site likelihood method for detecting positive selection at the molecular level. Mol. Biol. Evol.

[b95] Zhou Q, Bachtrog D (2012). Chromosome-wide gene silencing initiates Y degeneration in *Drosophila*. Curr. Biol.

