# Supplementary figures and images for: INDEPENDENT STRATUM FORMATION ON THE AVIAN SEX CHROMOSOMES REVEALS INTER-CHROMOSOMAL GENE CONVERSION AND PREDOMINANCE OF PURIFYING SELECTION ON THE W CHROMOSOME
Source: Evolution. 2014 Aug 29;68(11):3281–95. doi: 10.1111/evo.12493 (PMC4278454; doi:10.1111/evo.12493)

A.

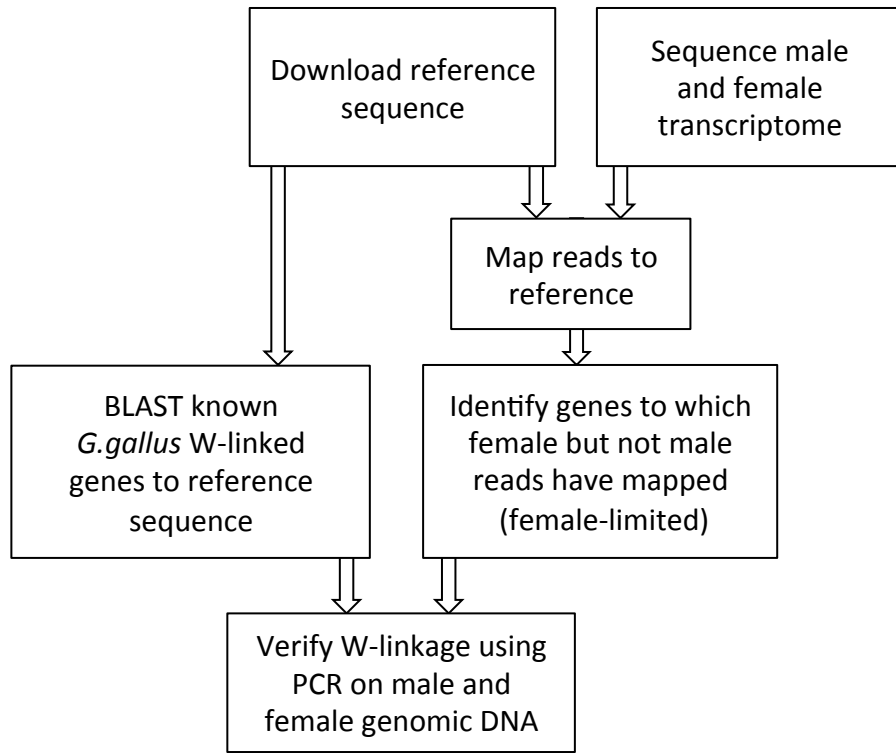

B.

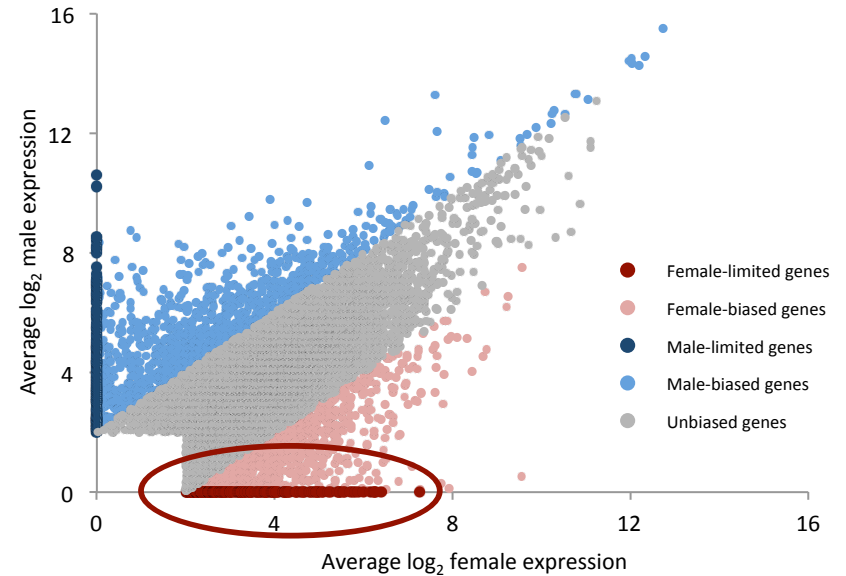

C.

Autosomal gene

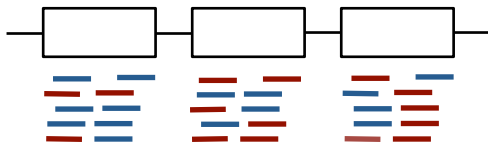

Z-linked gene

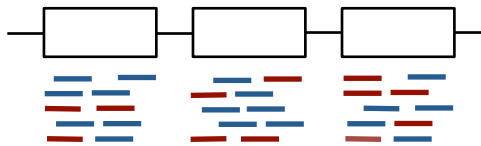

W-linked gene

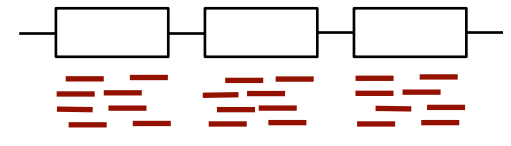

Male read  
Female read

Supplement: Figure S1 — Identification of W-linked genes. [file evo0068-3281-SD1.pdf]
