# Supplementary material for: INDEPENDENT STRATUM FORMATION ON THE AVIAN SEX CHROMOSOMES REVEALS INTER-CHROMOSOMAL GENE CONVERSION AND PREDOMINANCE OF PURIFYING SELECTION ON THE W CHROMOSOME
Source: Evolution. 2014 Aug 29;68(11):3281–95. doi: 10.1111/evo.12493 (PMC4278454; doi:10.1111/evo.12493)
Supplement: Figure S2 — Gene trees for M. gallopavo gametologs. [file evo0068-3281-SD2.pdf]

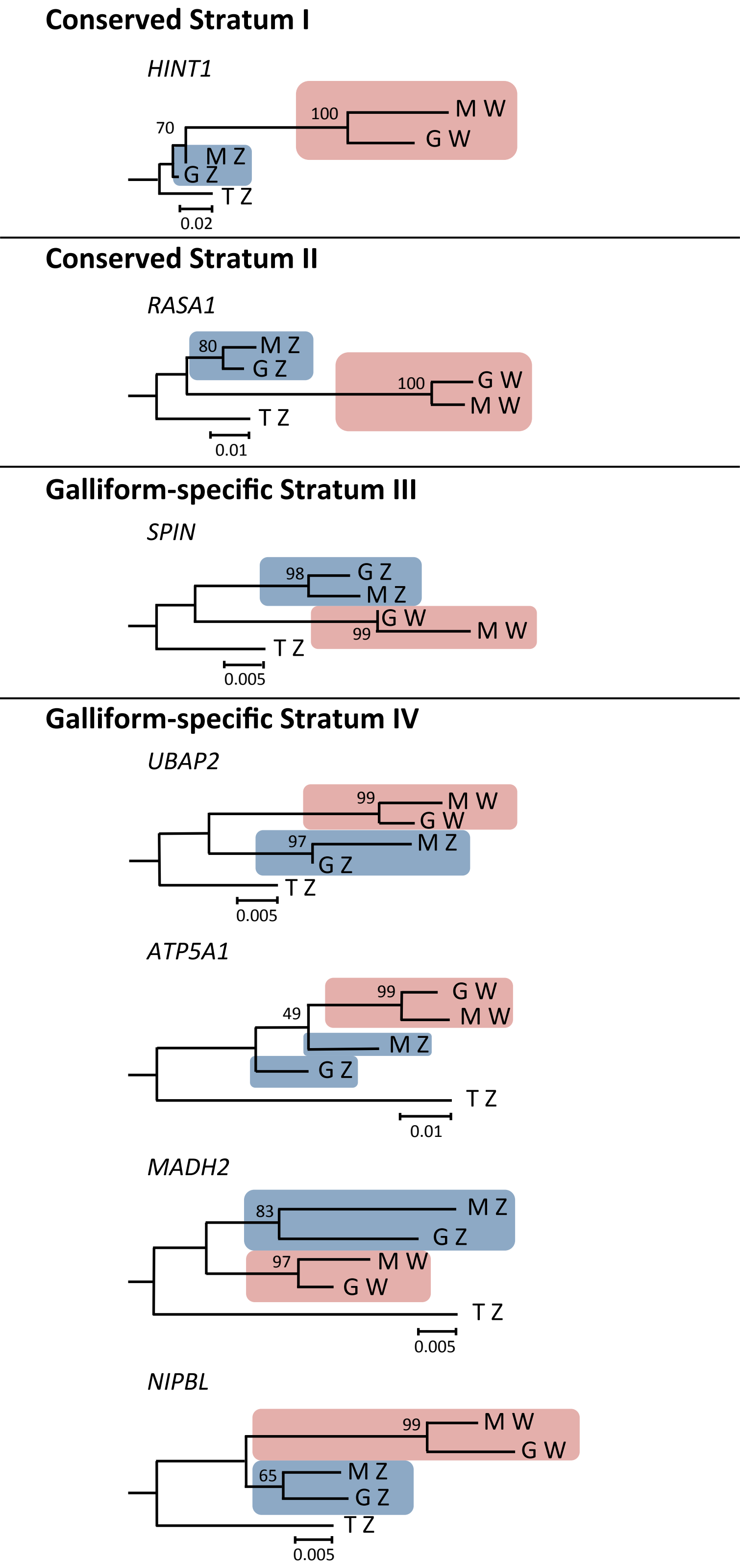
Conserved Stratum I
HINT1
70
100
M W
G W
M Z
G Z
T Z
0.02
Conserved Stratum II
RASA1
80
M Z
G Z
100
G W
M W
T Z
0.01
Galliform-specific Stratum III
SPIN
98
G Z
M Z
G W
99
M W
T Z
0.005
Galliform-specific Stratum IV
UBAP2
99
M W
G W
97
M Z
G Z
T Z
0.005
ATP5A1
99
G W
M W
49
M Z
G Z
T Z
0.01
MADH2
83
M Z
G Z
97
M W
G W
T Z
0.005
NIPBL
99
M W
G W
65
M Z
G Z
T Z
0.005
