# Supplementary material for: INDEPENDENT STRATUM FORMATION ON THE AVIAN SEX CHROMOSOMES REVEALS INTER-CHROMOSOMAL GENE CONVERSION AND PREDOMINANCE OF PURIFYING SELECTION ON THE W CHROMOSOME
Source: Evolution. 2014 Aug 29;68(11):3281–95. doi: 10.1111/evo.12493 (PMC4278454; doi:10.1111/evo.12493)

Conserved Stratum I

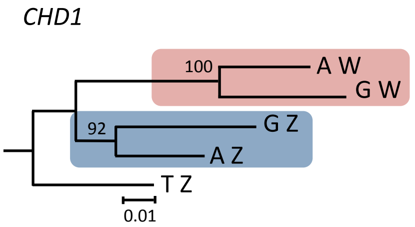

Conserved Stratum II

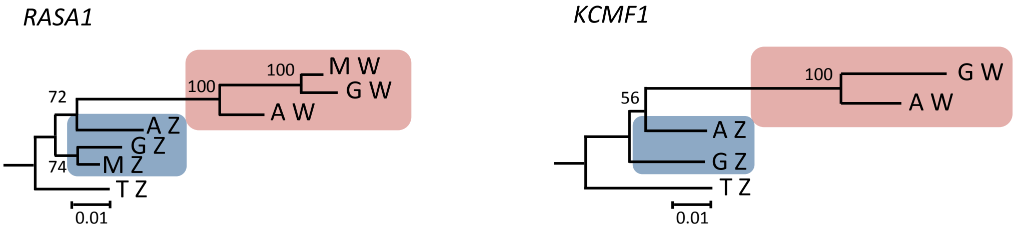

Anseriform-specific Stratum III

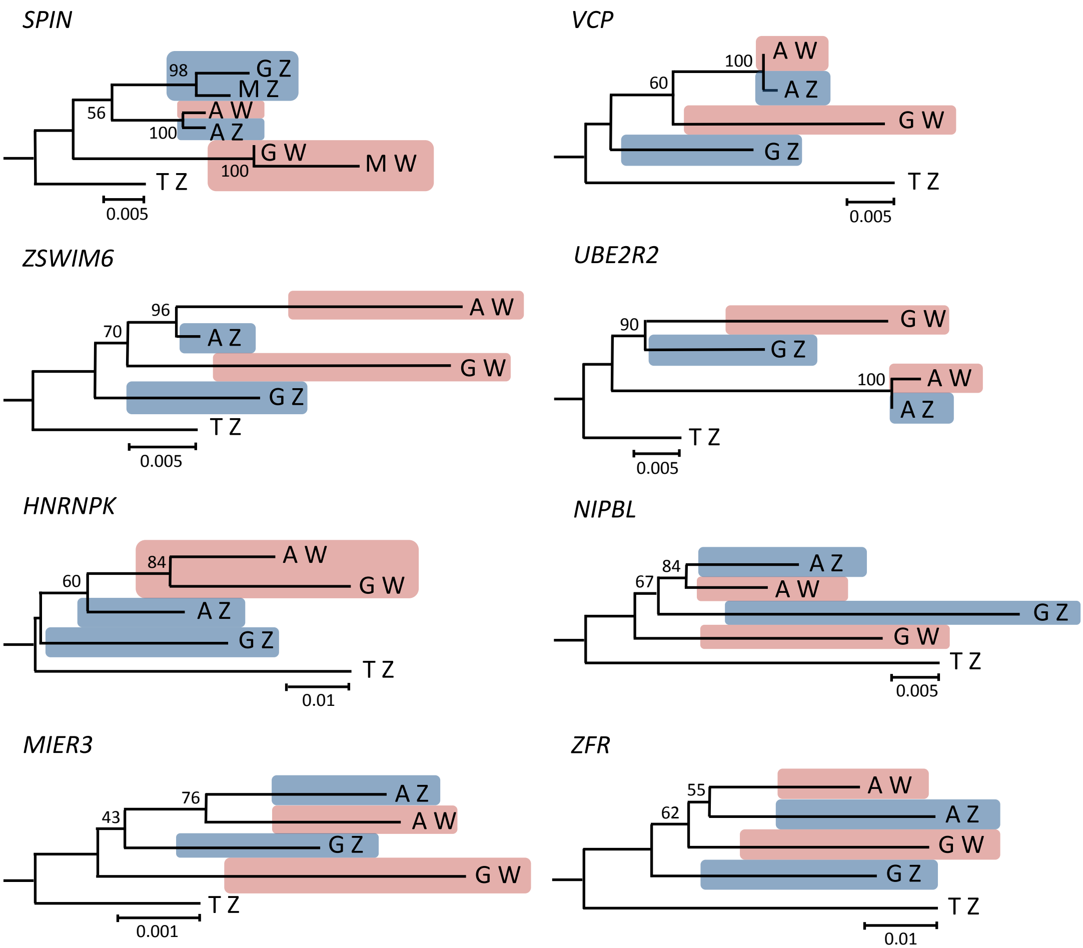

Supplement: Figure S3 — Gene trees for A. platyrhynchos gametologs. [file evo0068-3281-SD3.pdf]
