# Supplementary material for: INDEPENDENT STRATUM FORMATION ON THE AVIAN SEX CHROMOSOMES REVEALS INTER-CHROMOSOMAL GENE CONVERSION AND PREDOMINANCE OF PURIFYING SELECTION ON THE W CHROMOSOME
Source: Evolution. 2014 Aug 29;68(11):3281–95. doi: 10.1111/evo.12493 (PMC4278454; doi:10.1111/evo.12493)
Supplement: Table S1 — Sequence divergence between G. gallus gametologs. Table S2. Sequence divergence between M. gallopavo gametologs. Table S3. Sequence divergence between A. platyrhynchos gametologs. Table S4. Branch models and branch-site test across W-linked branches. Table S5. Branch models and branch-site test across Z-linked branches. [file evo0068-3281-SD4.pdf]

**Table S1. Sequence divergence between *G. gallus* gametologs**

| Gene pair (Z/W) | Gene ID[a] (Z/W) | Position on *G .gallus* Z (mb) | *G. gallus* $d_S$ (SE)[b] | *G.gallus* $d_N$ (SE)[b] | Putative stratum | Divergence (MYA) |
|---|---|---|---|---|---|---|
| *CHD1Z/ CHD1W* | 14642/ *CHDW*[1] | 51.10 | 0.390 (0.0246) | 0.0322 (0.0029) | Conserved I | 103 |
| *HINT1Z/ HINT1W* | 00428/ 22690 | 44.76 | 0.404 (0.1087) | 0.275 (0.0503) | Conserved I | 106 |
| *RASA1Z/ RASA1W* | 17706/ 22611 | 60.83 | 0.241 (0.0526) | 0.028 (0.0094) | Conserved II | 63 |
| *KCMF1Z/ KCMF1W* | 15391/ 14441 | 53.60 | 0.271 (0.0371) | 0.036 (0.0068) | Conserved II | 71 |
| *SPINZ/ SPINW* | 14916/ *SPINW*[1] | 43.03 | 0.178 (0.0347) | 0.007 (0.0035) | Galliform-specific III | 47 |
| *HNRNPKZ/ HNRNPKW* | 12591/ 14366 | 39.94 | 0.268 (0.0351) | 0.003 (0.0019) | Galliform-specific III | 71 |
| *BTF3Z/ BTF3W* | 13512/ 00395 | 24.60 | 0.245 (0.0526) | 0.024 (0.0087) | Galliform-specific III | 64 |
| *ZSWIM6Z/ ZSWIM6W* | 14734/ 27170 | 18.95 | 0.156 (0.0210) | 0.024 (0.0048) | Galliform-specific III | 41 |
| *MIER3Z/ MIER3W* | 14721/ 00140 | 17.22 | 0.210 (0.0426) | 0.025 (0.0077) | Galliform-specific III | 55 |
| *NIPBLZ/ NIPBLW* | 03605/ 13312 | 11.23 | 0.198 (0.0111) | 0.023 (0.0018) | Galliform-specific IV | 52 |
| *RPL17LZ/ RPL17LW* | 02696/ 22174 | 10.80 | 0.169 (0.0212) | 0.000 (0.0000) | Galliform-specific IV | 44 |
| *ZFRZ/ ZFRW* | 03235/ 14545 | 9.61 | 0.158 (0.0157) | 0.018 (0.0029) | Galliform-specific IV | 42 |
| *VCPZ/ VCPW* | 01986/ 00386 | 8.40 | 0.162 (0.0214) | 0.000 (0.0009) | Galliform-specific IV | 43 |
| *UBAP2Z/ UBAP2W* | 13809/ 05785 | 7.22 | 0.194 (0.0165) | 0.049 (0.0049) | Galliform-specific IV | 51 |
| *UBE2R2Z/ UBE2R2W* | 01668/ 09227 | 7.16 | 0.257 (0.0258) | 0.000 (0.0000) | Galliform-specific IV | 68 |
| *ATP5A1Z/ ATP5A1W* | 14644/ 01756 | 2.15 | 0.193 (0.0231) | 0.019 (0.0042) | Galliform-specific IV | 51 |
| *CZH18ORF25Z/ CZH18ORF25W* | 01763/ 01585 | 2.10 | 0.139 (0.0294) | 0.072 (0.0126) | Galliform-specific IV | 37 |
| *MADH2Z/ MADH2W* | 14697/ 10056 | 1.52 | 0.191 (0.0251) | 0.005 (0.0023) | Galliform-specific IV | 50 |
| *ZNF532Z/ ZNF532W* | 02852/ 14003 | 0.86 | 0.152 (0.0151) | 0.032 (0.0039) | Galliform-specific IV | 40 |
| *SIAT8CZ/ SIAT8CW* | 03049/ 26991 | 0.44 | 0.173 (0.0266) | 0.021 (0.0053) | Galliform-specific IV | 45 |

[a]Ensembl gene ID Ensgalg000000...
[b]Standard errors generated in Paml.
[1]Ayers et al (2013) Genome Biol

**Table S2. Sequence divergence between *M. gallopavo* gametologs**

| Gene pair (Z/W) | Gene ID[a] (Z/W) | Position on *G. gallus*/*M. gallopavo* Z (mb) | *M. gallopavo* $d_S$ (SE)[b] | *M. gallopavo* $d_N$ (SE)[b] | Putative stratum | Divergence (MYA) |
|---|---|---|---|---|---|---|
| *HINT1Z/* *HINT1W* | 06655/ 16898 | 44.76/47.46 | 0.285 (0.0961) | 0.182 (0.0434) | Conserved I | 75 |
| *RASA1Z/* *RASA1W* | 08292/ AH015047.1[1] | 60.83/64.54 | 0.266 (0.0600) | 0.0312 (0.0106) | Conserved II | 70 |
| *SPINZ/* *SPINW* | 02500/ AH015050.1[1] | 43.03/45.85 | 0.198 (0.0423) | 0.0129 (0.0054) | Galliform-specific III | 52 |
| *NIPBLZ/* *NIPBLW* | 01989/ 13606 | 11.23/12.01 | 0.182 (0.0453) | 0.0087 (0.0051) | Galliform-specific IV | 48 |
| *UBAP2Z/* *UBAP2W* | 01837/ AY188758.1[1] | 7.22/75.78 | 0.137 (0.0487) | 0.0273 (0.0139) | Galliform-specific IV | 36 |
| *ATP5A1Z/* *ATP5A1W* | 09963/ 01414 | 2.15/2.23 | 0.141 (0.0326) | 0.0076 (0.0044) | Galliform-specific IV | 37 |
| *MADH2Z/* *MADH2W* | 01351/ AH015049.1[1] | 1.52/1.51 | 0.146 (0.0485) | 0.0159 (0.0093) | Galliform-specific IV | 38 |

[a]Ensembl gene ID Ensmgag000000...
[b]Standard errors generated in Paml
[1]Berlin and Ellegren (2006) J Mol Ev

**Table S3. Sequence divergence between *A. platyrhynchos* gametologs**

| Gene pair (Z/W) | Gene ID[a] (Z/W) | Position on *G. gallus* Z (mb) | *A. platyrhynchos* $d_S$ (SE)[b] | *A. platyrhynchos* $d_N$ (SE)[b] | Putative stratum | Divergence (MYA) |
|---|---|---|---|---|---|---|
| *CHD1Z/ CHD1W* | 09965/ 05191 | 51.10 | 0.296 (0.0399) | 0.0317 (0.0057) | Conserved I | 78 |
| *RASA1Z/ RASA1W* | 05627/ 05611 | 60.83 | 0.171 (0.0346) | 0.008 (0.0039) | Conserved II | 45 |
| *KCMF1Z/ KCMF1W* | 13426/ 03026 | 53.60 | 0.198 (0.0343) | 0.0371 (0.0081) | Conserved II | 52 |
| *SPINZ/ SPINW* | 13922/ 02923 | 43.03 | 0.014* (0.0000) | 0.000 (0.0000) | Anseriform- specific III | 4 |
| *HNRNPKZ/ HNRNPKW* | 10856/ 10986 | 39.94 | 0.148 (0.0249) | 0.005 (0.0023) | Anseriform-specific III | 39 |
| *ZSWIM6Z/ ZSWIM6W* | 06992/ 13555 | 19.00 | 0.065* (0.0153) | 0.0131 (0.0045) | Anseriform-specific III | 17 |
| *MIER3Z/ MIER3W* | 06634/ 010850 | 17.22 | 0.104 (0.0182) | 0.0278 (0.0055) | Anseriform-specific III | 27 |
| *NIPBLZ/ NIPBLW* | 08473/ 10290 | 11.23 | 0.077* (0.0204) | 0.0017 (0.0017) | Anseriform-specific III | 20 |
| *ZFRZ/ ZFRW* | 15627/ 15519 | 9.61 | 0.133 (0.0264) | 0.0256 (0.0062) | Anseriform-specific III | 35 |
| *VCPZ/ VCPW* | 04533/ 05806 | 8.40 | 0.005* (0.0000) | 0.000 (0.0000) | Anseriform-specific III | 1 |
| *UBE2R2Z/ UBE2R2W* | 03800/ 16000 | 1.76 | 0.000* (0.0000) | 0.004 (0.0042) | Anseriform-specific III | 0 |

[a]Ensembl gene ID Ensaplg000000...

[b]Standard errors generated in Paml

*Significantly different from *G. gallus* $d_S$ estimates (non-overlapping 95% confidence intervals)

**Table S4. Branch models and branch-site test across W-linked branches**

| | | | | | Branch model (ω estimated) | | Branch model test (ω =1) | | Branch model test (ω =0) | | Branch-site test (ω =1) | |
|---|---|---|---|---|---|---|---|---|---|---|---|---|
| Gene name | *G. gallus* Ensembl ID (Z/W)[a] | *A. platyrhynchos* Ensembl ID (Z/W)[b] | *M. gallopavo* Ensembl ID (Z/W)[c] | *T. guttata* Ensembl ID (Z)[d] | -lnL | ω | −lnL | LR | −lnL | LR | −lnL (ω estimated) | −lnL (ω =1) |
| *HINT1* | 00428/22690 | - | 06655/16898 | 00395 | -365.13 | 1.10 | -365.15 | 0.05 | -2213.65 | **3697.05**** | -362.45 | -362.47 |
| *CHD1* | 14642/*CHDW*[1] | 09965/05191 | - | 01185 | -3027.39 | 0.14 | -3074.62 | **94.45**** | -6533.47 | **7012.15**** | -3009.74 | -3009.74 |
| *RASA1* | 17706/22611 | 05611/05627 | 08292/AH015047.1[2] | 01169 | -697.16 | 0.09 | -712.82 | **31.33**** | -1390.63 | **1386.95**** | -697.08 | -697.08 |
| *KCMF1* | 15391/14441 | 13426/03026 | - | 00265 | -1746.53 | 0.22 | -1764.27 | **35.49**** | -4583.88 | **5674.71**** | -1736.81 | -1736.92 |
| *MIER3* | 14721/00140 | 06634/10850 | - | 02601 | -1292.61 | 0.24 | -1301.60 | **17.97**** | -1376.24 | **167.25**** | -1293.55 | -1293.55 |
| *SPIN* | 14916/*SPINW*[1] | 13922/02923 | 02500/AH015050.1[2] | 00468 | -1153.90 | 0.09 | -1168.12 | **28.45**** | -2027.39 | **1749.98**** | -1154.67 | -1154.67 |
| *HNRNPK* | 12591/14366 | 10856/10986 | - | 03138 | -2340.51 | 0.03 | -2394.89 | **108.75**** | -2382.62 | **84.21**** | -2332.91 | -2332.92 |
| *VCP* | 01986/00386 | 04533/05806 | - | 01760 | -1081.34 | 0.03 | -1094.13 | **25.58**** | -1090.90 | **19.13**** | -1081.23 | -1081.31 |
| *ZFR* | 03235/14545 | 15627/15519 | - | 01909 | -1973.85 | 0.18 | -1987.42 | **27.16**** | -2060.59 | **173.50**** | -1963.95 | -1963.95 |
| *NIPBL* | 03605/13312 | 08473/10290 | - | 02047 | -1487.07 | 0.05 | -1509.10 | **44.08**** | -1519.02 | **63.91**** | -1487.31 | -1487.31 |
| *NIPBL* | 03605/13312 | - | 13606/01989 | 02047 | -838.30 | 0.03 | -857.50 | **38.40**** | -1010.05 | **343.5**** | -838.37 | -838.37 |
| *UBAP2* | 13809/05785 | - | 01837/AY188758.1[2] | 01689 | -406.73 | 0.15 | -409.83 | **6.19*** | -584.14 | **354.82**** | -406.74 | -406.74 |
| *ATP5A1* | 14644/01756 | - | 09963/01414 | 01582 | -1101.40 | 0.09 | -1109.67 | **16.54**** | -1426.82 | **650.83**** | -1096.96 | -1098.75 |
| *MADH2* | 14697/14184 | - | 01351/AH015049.1[2] | 00016 | -1151.96 | 0.06 | -1163.14 | **22.37**** | -1318.71 | **33.50**** | -1149.58 | -1149.58 |
| *UBE2R2* | 01668/09227 | 03800/16000 | - | 01683 | -514.68 | 0.04 | -522.19 | **15.03**** | -523.37 | **17.39**** | -514.68 | -514.68 |
| *ZSWIM6* | 06992/13555 | 14734/27170 | - | 02716 | -1014.36 | 0.25 | -1019.98 | **11.25**** | -1078.80 | **128.88**** | -1014.15 | -1014.15 |

[a]Ensgalg000000...
[b]Ensaplg000000...
[c]Ensmgag000000...
[d]Enstgug000000...
** p-value <0.01
* p-value <0.05
[1]Ayers et al (2013) Genome Biol
[2]Berlin and Ellegren (2006) J Mol Ev

**Table S5. Branch models and branch-site test across Z-linked branches**

| Gene name | *G. gallus* Ensembl ID (Z/W)[a] | *A. platyrhynchos* Ensembl ID (Z/W)[b] | *M.gallopavo* Ensembl ID (Z/W)[c] | *T. guttata* Ensembl ID (Z)[d] | Branch model (ω estimated) | | Branch model test (ω =1) | | Branch model test (ω =0) | | Branch-site test (ω =1) | |
|---|---|---|---|---|---|---|---|---|---|---|---|---|
| | | | | | −lnL | ω | −lnL | LR | −lnL | LR | −lnL (ω estimated) | −lnL (ω =1) |
| *HINT1* | 00428/22690 | - | 06655/16898 | 00395 | -367.32 | 0.00 | -370.99 | **7.34**** | -367.32 | 0.00 | -370.17 | -370.17 |
| *CHD1* | 14642/*CHDW*[1] | 09965/05191 | - | 01185 | -3035.79 | 0.03 | -3122.01 | **172.44**** | -4005.27 | **1938.96**** | -3026.57 | -3026.57 |
| *RASA1* | 17706/22611 | 05611/05627 | 08292/AH015047.1[2] | 01169 | -696.13 | 0.03 | -709.51 | **26.77**** | -868.30 | **344.34**** | -697.08 | -697.08 |
| *KCMF1* | 15391/14441 | 13426/03026 | - | 00265 | -1749.50 | 0.12 | -1764.45 | **29.89**** | -3019.36 | **259.71**** | -1740.93 | -1740.93 |
| *MIER3* | 14721/00140 | 06634/10850 | - | 02601 | -1293.87 | 0.11 | -1306.99 | **26.23**** | -1324.28 | **60.82**** | -1294.25 | -1294.25 |
| *SPIN* | 14916/*SPINW*[1] | 13922/02923 | 02500/AH015050.1[2] | 00468 | -1156.96 | 0.02 | -1172.79 | **31.65**** | -1326.73 | **339.55**** | -1157.20 | -1157.20 |
| *HNRNPK* | 12591/14366 | 10856/10986 | - | 03138 | -2339.38 | 0.01 | -2393.62 | **108.49**** | -2343.99 | **9.22**** | -2333.75 | -2333.75 |
| *VCP* | 01986/00386 | 04533/05806 | - | 01760 | -1081.20 | 0.00 | -1095.52 | **28.65**** | -1081.20 | 0.00 | -1081.67 | -1081.67 |
| *ZFR* | 03235/14545 | 15627/15519 | - | 01909 | -1975.63 | 0.12 | -2003.04 | **54.84**** | -2055.82 | **160.38**** | -1968.12 | -1968.16 |
| *NIPBL* | 03605/13312 | 08473/10290 | - | 02047 | -1487.64 | 0.02 | -1529.38 | **83.48**** | -1503.87 | **32.46**** | -1488.06 | -1488.06 |
| *NIPBL* | 03605/13312 | - | 13606/01989 | 02047 | -838.46 | 0.04 | -845.81 | **14.70**** | -1008.86 | **340.80**** | -838.47 | -838.47 |
| *UBAP2* | 13809/05785 | - | 01837/AY188758.1[2] | 01689 | -406.59 | 0.25 | -407.82 | 2.47 | -751.58 | **689.98**** | -406.61 | -406.61 |
| *ATP5A1* | 14644/01756 | - | 09963/01414 | 01582 | -1105.90 | 0.00 | -1132.66 | **53.53**** | -1105.89 | -0.01 | -1103.10 | -1103.10 |
| *MADH2* | 14697/14184 | - | 01351/AH015049.1[2] | 00016 | -1152.46 | 0.02 | -1182.98 | **61.03**** | -1485.78 | **666.65**** | -1150.21 | -1150.21 |
| *UBE2R2* | 01668/09227 | 03800/16000 | - | 01683 | -515.67 | 0.00 | -520.67 | **10.01**** | -515.66 | 0.00 | -515.82 | -515.82 |
| *ZSWIM6* | 06992/13555 | 14734/27170 | - | 02716 | -1014.55 | 0.05 | -1021.05 | **12.99**** | -1020.57 | **12.05**** | -1015.06 | -1015.06 |

[a]Ensgalg000000...
[b]Ensaplg000000...
[c]Ensmgag000000...
[d]Enstgug000000...
** p-value <0.01
* p-value <0.05
[1]Ayers et al (2013) Genome Biol
[2]Berlin and Ellegren (2006) J Mol Evol
