# Supplementary material for: INDEPENDENT STRATUM FORMATION ON THE AVIAN SEX CHROMOSOMES REVEALS INTER-CHROMOSOMAL GENE CONVERSION AND PREDOMINANCE OF PURIFYING SELECTION ON THE W CHROMOSOME
Source: Evolution. 2014 Aug 29;68(11):3281–95. doi: 10.1111/evo.12493 (PMC4278454; doi:10.1111/evo.12493)
Supplement: Supplementary file 5 — Supplemental Figure Legends [file evo0068-3281-SD5.docx]

**Supplemental Figure 1: Identification of W-linked genes**

Panel A. Pipeline of expression and BLAST based approaches used to identify W-linked genes. Panel B. Schematic illustrating the distribution of sex-biased genes after Illumina reads are mapped to the reference genome. Female-limited genes and strongly female biased genes are highlighted as putative W-linked genes. Panel C. Diagram outlining the distribution of male and female Illumina reads after mapping to the reference genome. Female but not male reads will map to putative W-linked genes.

**Supplemental Figure 2: Gene trees for *M. gallopavo* gametologs**

Maximum-likelihood gene phylogenies for *M. gallopavo* gametologs are shown. Bootstrap values were calculated using 1000 permutations. AZ, GZ, MZ, TZ corresponds to *A. platyrhynchos*, *G. gallus*, *M. gallopavo* and *T. guttata* Z-linked genes.

**Supplemental Figure 3: Gene trees for *A. platyrhynchos* gametologs**

Maximum-likelihood gene phylogenies for *A. platyrhynchos* gametologs are shown. Bootstrap values were calculated using 1000 permutations. AZ, GZ, MZ, TZ corresponds to *A. platyrhynchos*, *G. gallus*, *M. gallopavo* and *T. guttata* Z-linked genes.
